# Nicotine Pouch Sales Trends in the US by Volume and Nicotine Concentration Levels From 2019 to 2022

**DOI:** 10.1001/jamanetworkopen.2022.42235

**Published:** 2022-11-15

**Authors:** Anuja Majmundar, Christian Okitondo, Ashley Xue, Samuel Asare, Priti Bandi, Nigar Nargis

**Affiliations:** 1 Tobacco Control Research, American Cancer Society Inc, Kennesaw, Georgia; 2 The University of Georgia, College of Public Health, Atlanta; 3 Risk Factors and Screening Surveillance Research, American Cancer Society Inc, Kennesaw, Georgia

## Abstract

This cross-sectional study examines the sale of 4 nicotine pouch brands in the contiguous 48 states and Washington, DC.

## Introduction

Nicotine pouches, dissolvable microfiber pouches prefilled with nicotine salt powder but not containing tobacco leaf, are gaining popularity in the US.^1^ Nicotine concentration levels in these products are comparable to snus and moist snuff^2^ and often exceed levels found in nicotine replacement therapy products,^3^ creating the potential for nicotine dependency instead of quitting among individuals who use nicotine and nicotine initiation or experimentation among those who are nicotine-naive.^4^ In this study, we assessed US nicotine pouch unit sales trends by volume and nicotine concentration levels.

## Methods

We analyzed data comprising weekly NielsenIQ Retail Scanner point-of-purchase sales from August 10, 2019, through March 26, 2022, for 2182 local trade areas in the contiguous 48 states and Washington, DC. This cross-sectional study was based on data that were not from human participants; thus, the US Department of Health and Human Services considered this study to be nonhuman research, which is exempt from review or informed consent. We followed the STROBE reporting guideline.

Four nicotine pouch brands (Zyn [Swedish Match], Rogue [Rogue Holdings], On! [Helix Innovations], and Velo [British American Tobacco]) were identified through a review of product characteristics in the sales data and market research. One unit was defined as 1 pouch given that the number of pouches per container varied (ranging from 12-20) across brands. Unit sales were aggregated by month and year overall and by brand and nicotine concentration level per unit. Shares of unit sales by brand and nicotine concentration level were calculated as a proportion of total unit sales.

Average monthly percentage change (AMPC) in unit sales and 95% CIs were calculated using Joinpoint, version 4.9.1.0 (National Cancer Institute), a segmented regression analysis application. Statistically significant changes included those with 95% CIs that did not cross 0. Two-sided α<.05 indicated significance.

## Results

**Figure 1.**
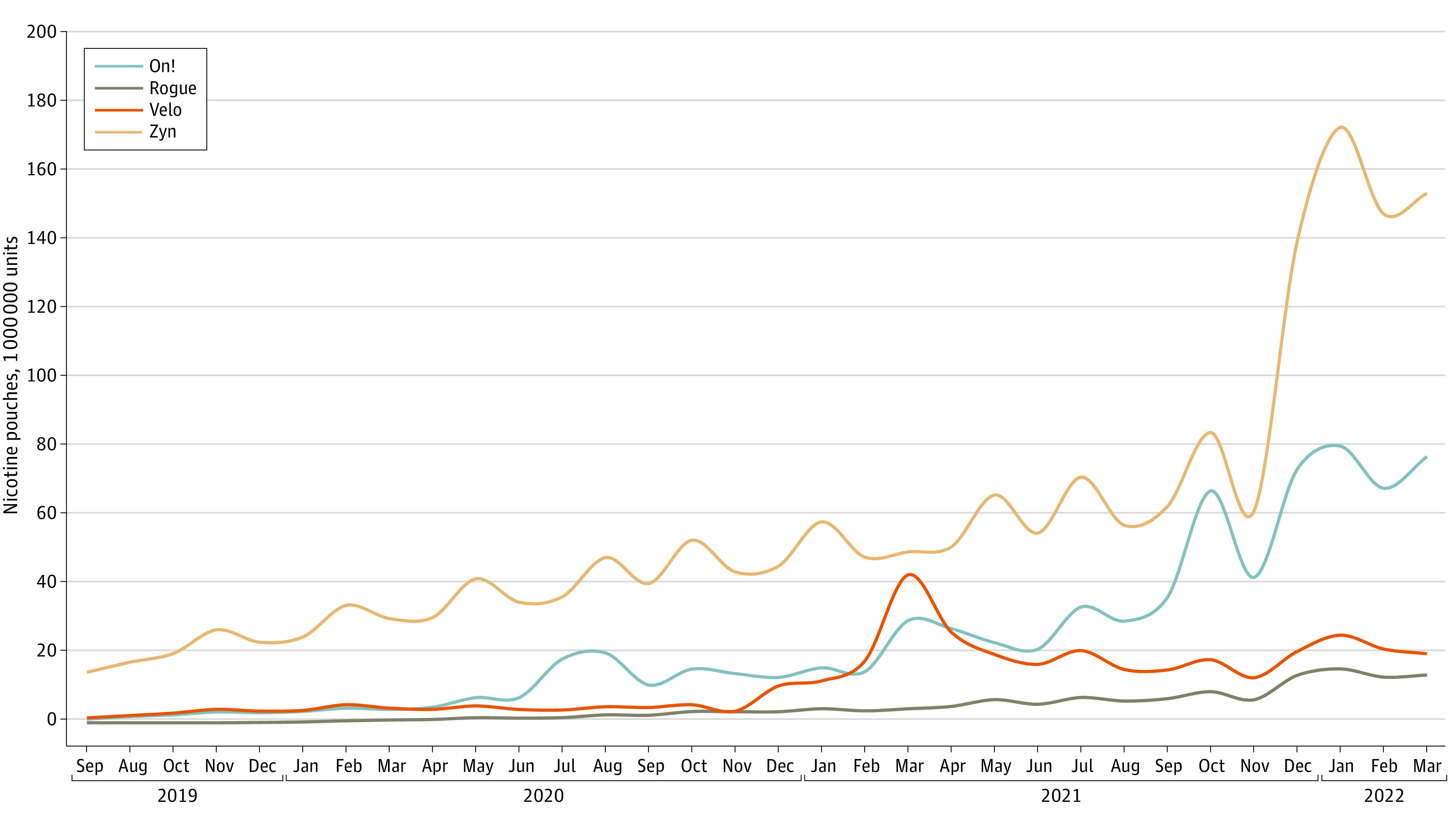
Nicotine Pouch Unit Sales in the US by Brand From 2019 to 2022 One unit represented 1 nicotine pouch. Rogue nicotine pouches were sold in the US starting November 2019. Sales of Velo nicotine pouches included Velo Max products.

Overall sales increased from 126.06 million units from August to December 2019 to 808.14 million units from January to March 2022 (AMPC, 8.1; 95% CI, 7.4-8.9) (Figure 1). Zyn led the overall unit share (58.8%), followed by On! (24.6%), Velo (12.1%), and Rogue (4.8%) during the study period (Figure 1). Zyn sales peaked in September 2021 and increased more than other brands from October 2021 to March 2022 (AMPC, 18.0; 95% CI, 8.9-27.8). However, Rogue sales increased more rapidly than all other brands (AMPC, 37.0; 95% CI, 31.3-42.9).

**Figure 2.**
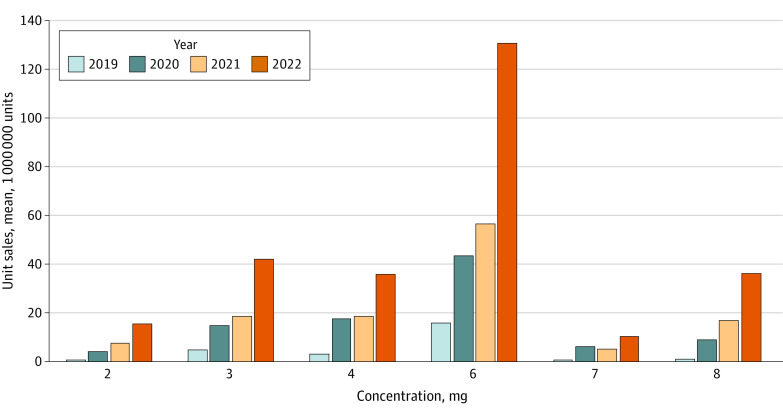
Mean Monthly Unit Sales of Nicotine Pouches in the US by Nicotine Concentration Levels From 2019 to 2022 One unit represented 1 nicotine pouch. Mean monthly unit sales were estimated from annual sales divided by months of data available for a given year. Nicotine pouches with 2 mg concentration levels were offered by On! and Velo; 3 mg, by On! and Rogue; 4 mg, by On! and Velo; 6 mg, by Rogue and Zyn; and 8 mg, by On!. Approximately 0.3% of total units did not include nicotine concentration levels.

Nicotine pouches with 6 mg (1365.19 million units), 4 mg (470.36 million units), and 3 mg (449.61 million units) nicotine concentration levels were most commonly sold during the study period. Sales of products with 8 mg nicotine concentration level (AMPC, 17.6; 95% CI, 13.8-21.6) increased more rapidly than products with lower concentration levels (2 mg: AMPC, 12.3 [95% CI, 10.9-13.7]; 3mg: APMC, 7.9 [95% CI, 6.1-9.7]; 4 mg: AMPC, 9.4 [95% CI, 7.3-11.6]; 6 mg: AMPC, 8.6 [95% CI, 6.1-11.1]; 7 mg: AMPC, 9.7 [95% CI, 4.9-14.6]) (Figure 2).

## Discussion

Similar to nicotine pouch sales trends from 2016 to 2020,^1^ the trajectory of nicotine pouch sales continued to rise through March 2022. Increasing sales of products with the highest (8 mg) nicotine concentration level raise concerns about abuse liability among individuals who use nicotine. Increasing sales of products with the lowest (2 mg) nicotine concentration level found in this study combined with higher sales of youth-appealing fruit flavors reported previously^1^ warrant continued surveillance of the uptake or experimentation trends among tobacco-naive youth. Sales trends are not generalizable to use patterns. A study limitation was that the data did not represent all nicotine pouch products and online sales in the US and did not account for intraproduct variation in nicotine concentration levels.

Nicotine pouch promotions highlight youth-appealing flavors and convenience of using these products anywhere. No manufacturer has received authorization from the US Food and Drug Administration to market nicotine pouches as a tobacco product or a cessation drug.^5,6^ Health campaigns warning of potential adverse health outcomes of nicotine pouches are needed.
